# Supplementary material for: Brief Oxygen Exposure after Traumatic Brain Injury Hastens Recovery and Promotes Adaptive Chronic Endoplasmic Reticulum Stress Responses
Source: Int J Mol Sci. 2023 Jun 6;24(12):9831. doi: 10.3390/ijms24129831 (PMC10298247; doi:10.3390/ijms24129831)
Supplement: Supplementary file 1 [file ijms-24-09831-s001.zip › Supplemental File_Western Blots.pdf]

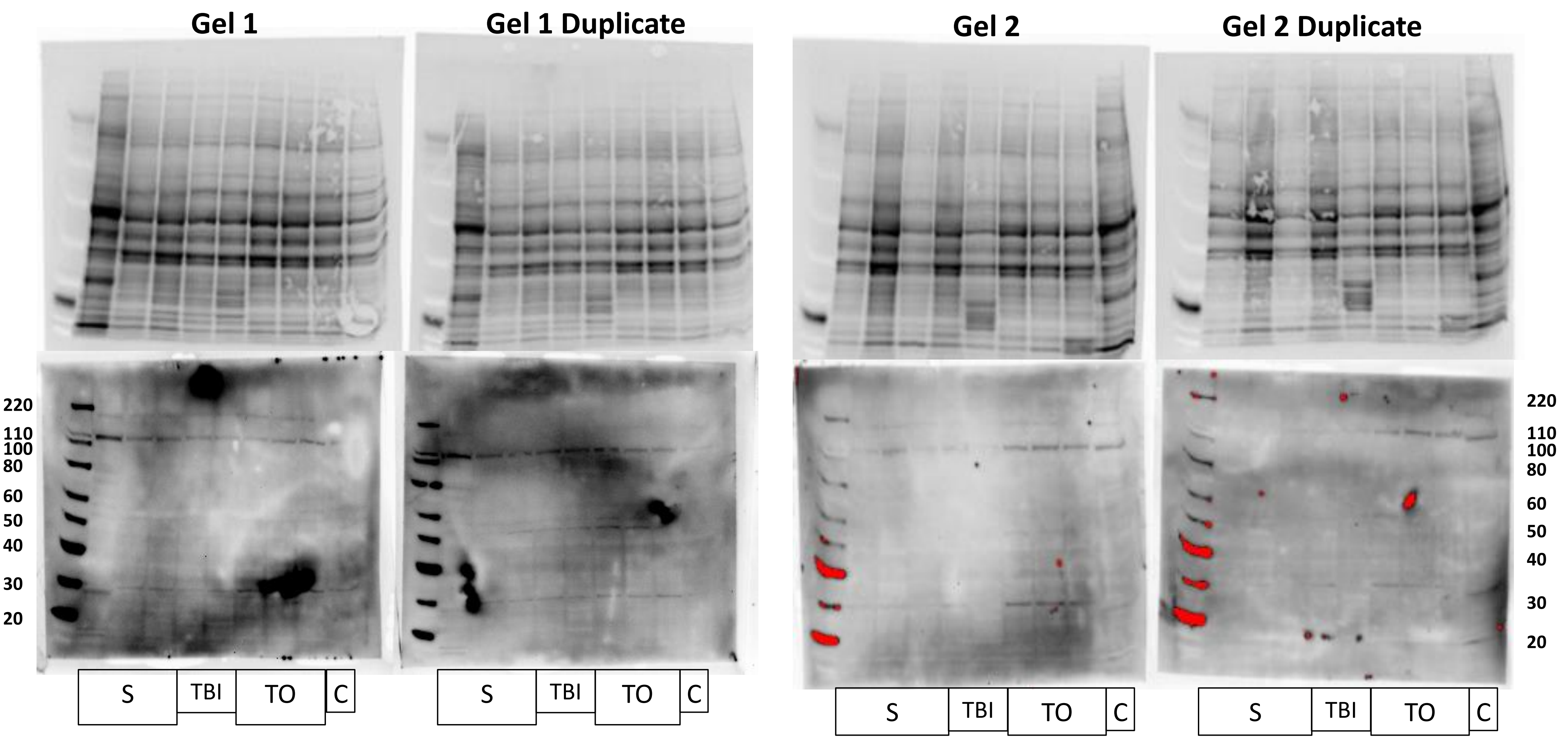

Raw Western Blot Membranes for IRE1 7 DPI

Bands obscured by anything we not included in analysis. Duplicates were averaged. Intermembrane calculations were done as described in our previous publication[13]. Top blot = Total Protein, Bottom Blot = Chemiluminescent image of primary antibody (IRE1 = ~110kDa). Ladder = Magic Mark. C = Intermembrane Control, S=SHAM, TBI = Room Air Group, TO = TBI +O_2

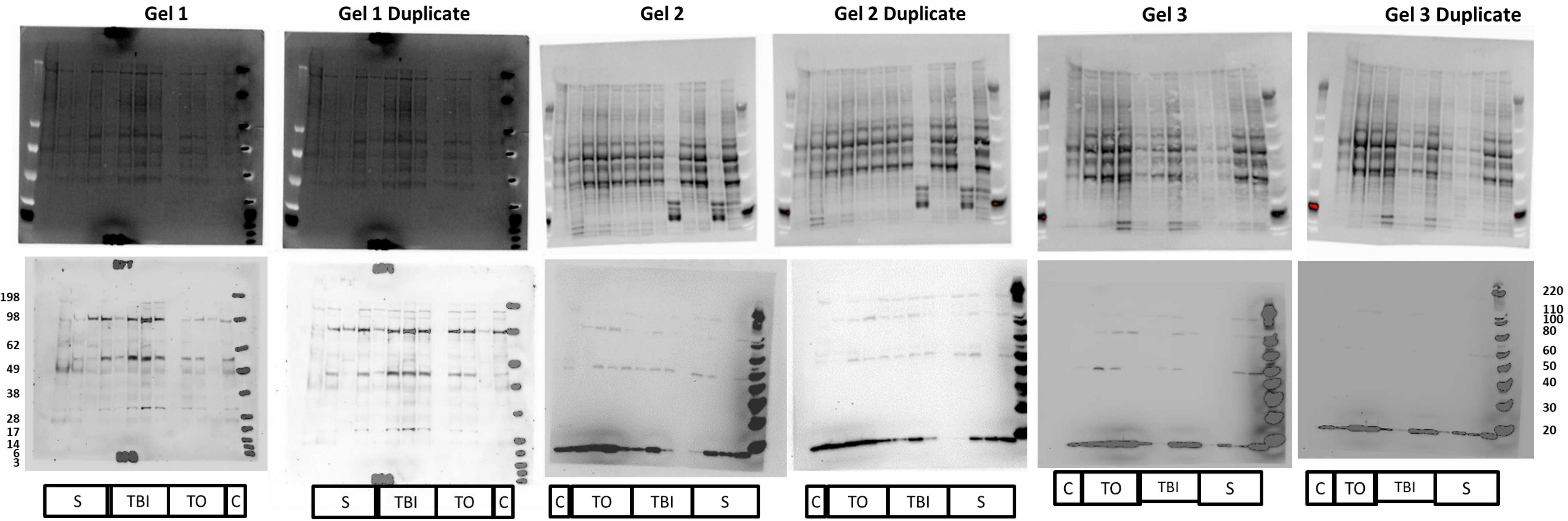

Raw Western Blot Membranes for IRE1 30 DPI

Bands obscured by anything we not included in analysis. Duplicates were averaged. Intermembrane calculations were done as described in our previous publication[13]. Top blot = Total Protein, Bottom Blot = Chemiluminescent image of primary antibody (IRE1 = ~110kDa). Ladder = SeeBluePlus2 or Magic Mark. C = Intermembrane Control, H = H_2O (negative), S=SHAM, TBI = Room Air Group, TO = TBI +O_2

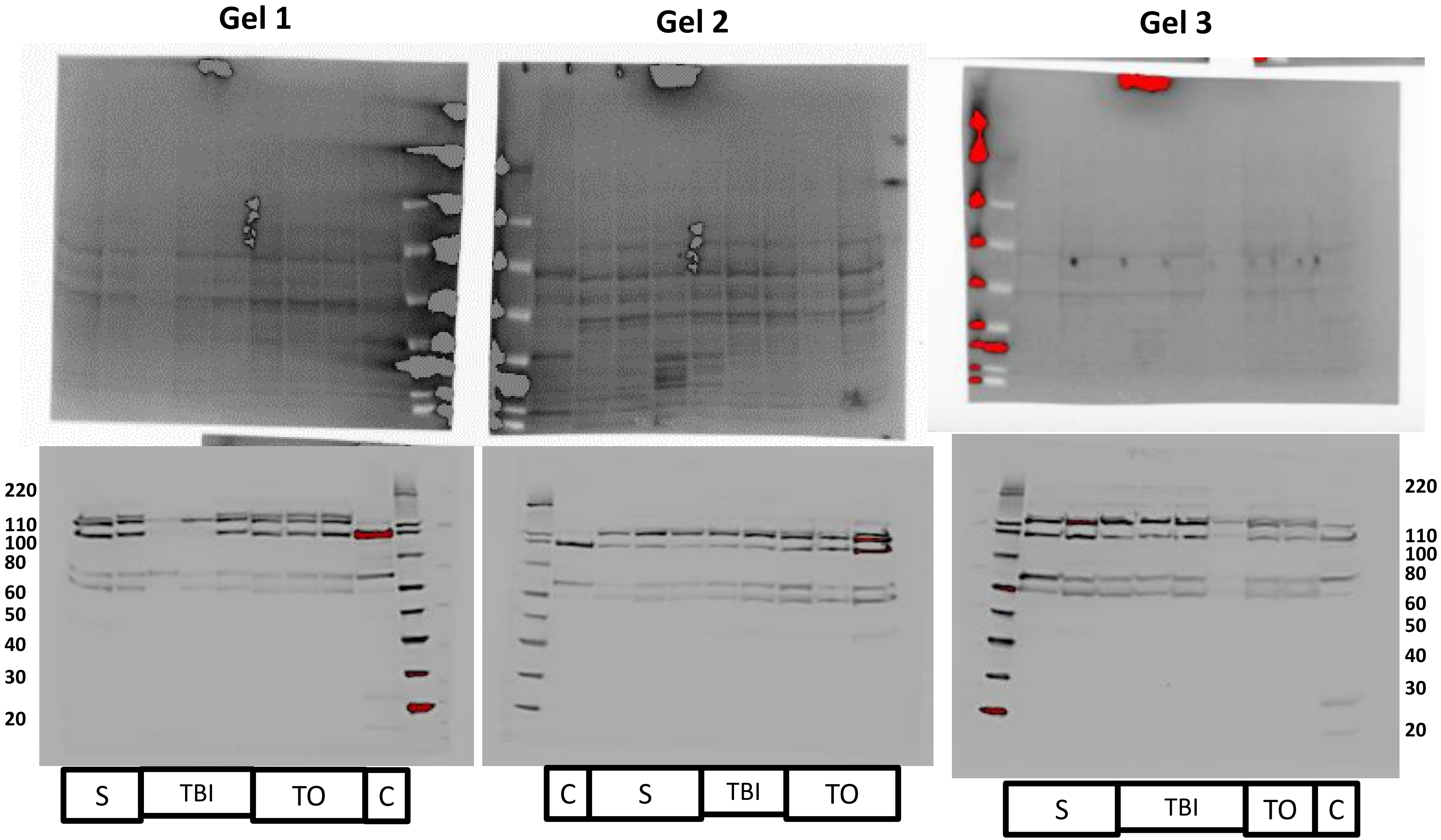

Raw Western Blot Membranes for XBP1-spliced 7 DPI

Bands obscured by anything we not included in analysis. Intermembrane calculations were done as described in our previous publication[13]. Protein Quantity was limited, so markers with higher abundance were not run in duplicate. Top blot = Total Protein, Bottom Blot = Chemiluminescent image of primary antibody (XBP1s = ~60kDa) Ladder = Magic Mark. C = Intermembrane Control, S= SHAM, TBI = Room Air Group, TO = TBI +O_2

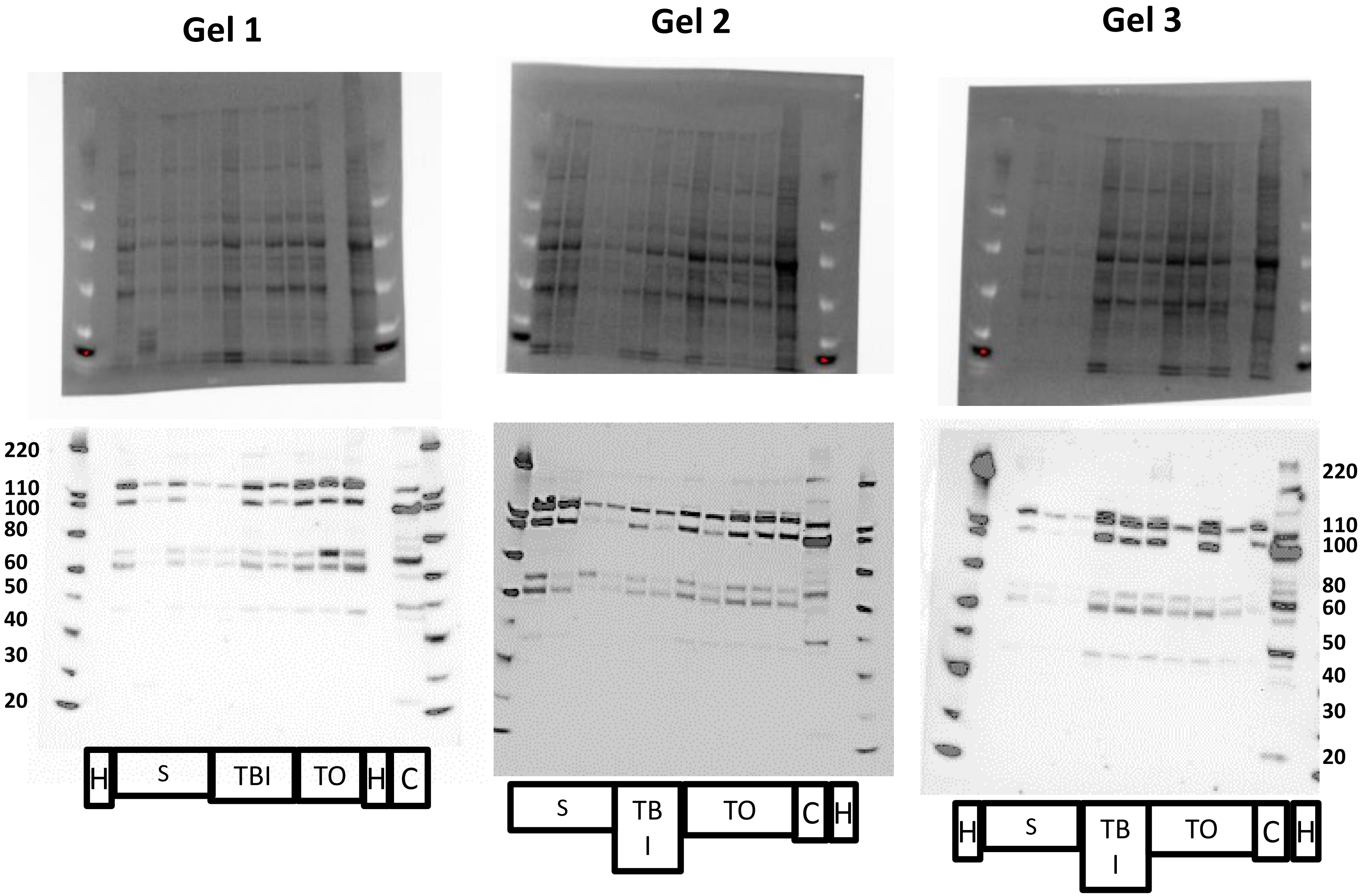

Raw Western Blot Membranes for XBP1-spliced 30 DPI

Bands obscured by anything we not included in analysis. Intermembrane calculations were done as described in our previous publication[13]. Protein Quantity was limited, so markers with higher abundance were not run in duplicate. Top blot = Total Protein, Bottom Blot = Chemiluminescent image of primary antibody (XBP1s = ~60kDa). Ladder = Magic Mark. C = Intermembrane Control, H = H_2O (negative), S=SHAM TBI = Room Air Group, TO = TBI +O_2

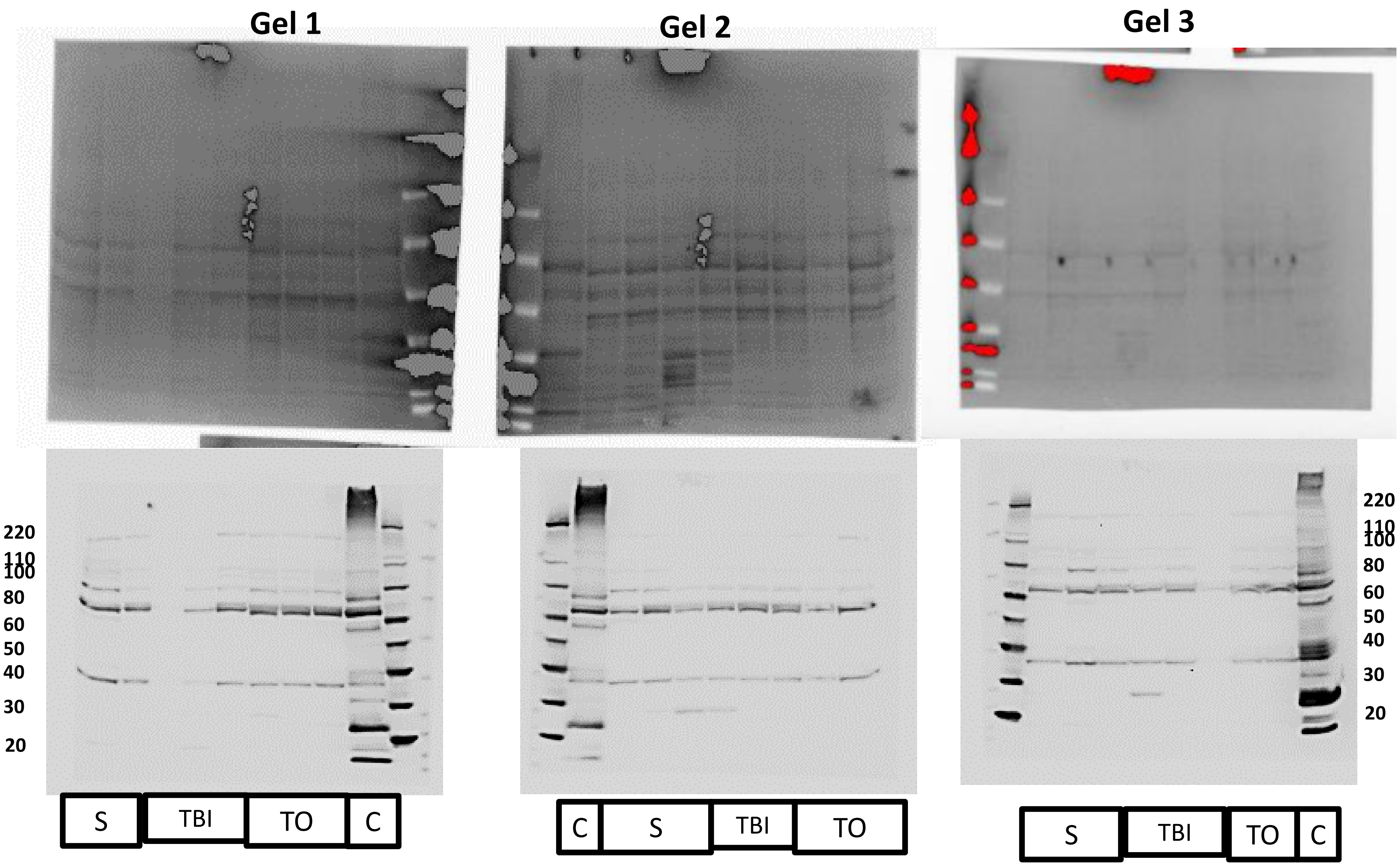

Raw Western Blot Membranes for XBP1-Unspliced7 DPI

Bands obscured by anything we not included in analysis. Intermembrane calculations were done as described in our previous publication[13]. Protein Quantity was limited, so markers with higher abundance were not run in duplicate. Top blot = Total Protein, Bottom Blot = Chemiluminescent image of primary antibody (XBP1U = ~38kDa). Ladder = Magic Mark. C = Intermembrane Control, H = H_2O (negative), S=SHAM, TBI = Room Air Group, TO = TBI +O_2

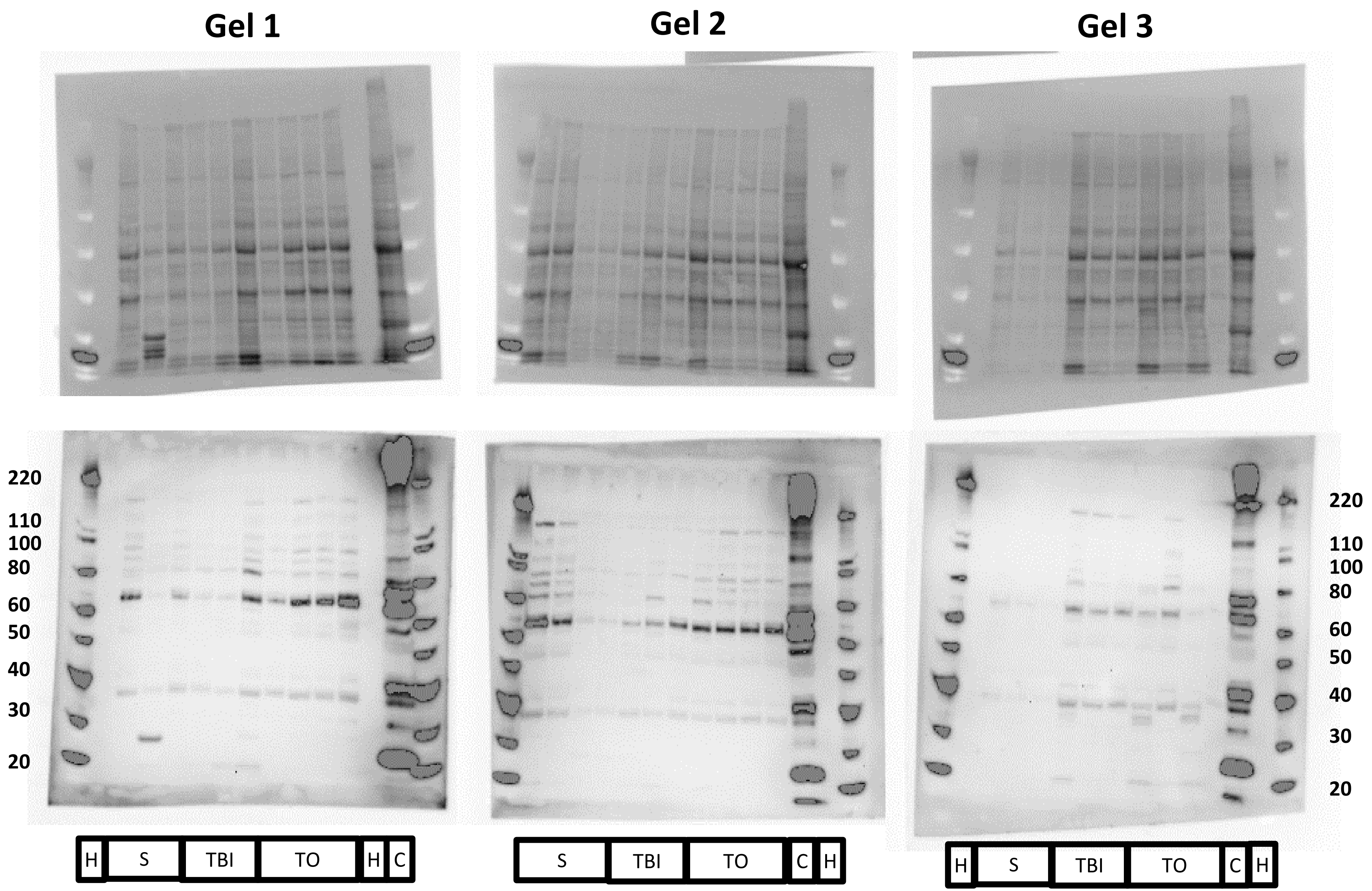

Raw Western Blot Membranes for XBP1-Unspliced 30 DPI

Bands obscured by anything we not included in analysis. Intermembrane calculations were done as described in our previous publication[13]. Protein Quantity was limited, so markers with higher abundance were not run in duplicate. Top blot = Total Protein, Bottom Blot = Chemiluminescent image of primary antibody (XBP1U = ~38kDa). Ladder = Magic Mark. C = Intermembrane Control, H = H_2O (negative), S=SHAM, TBI = Room Air Group, TO = TBI +O_2

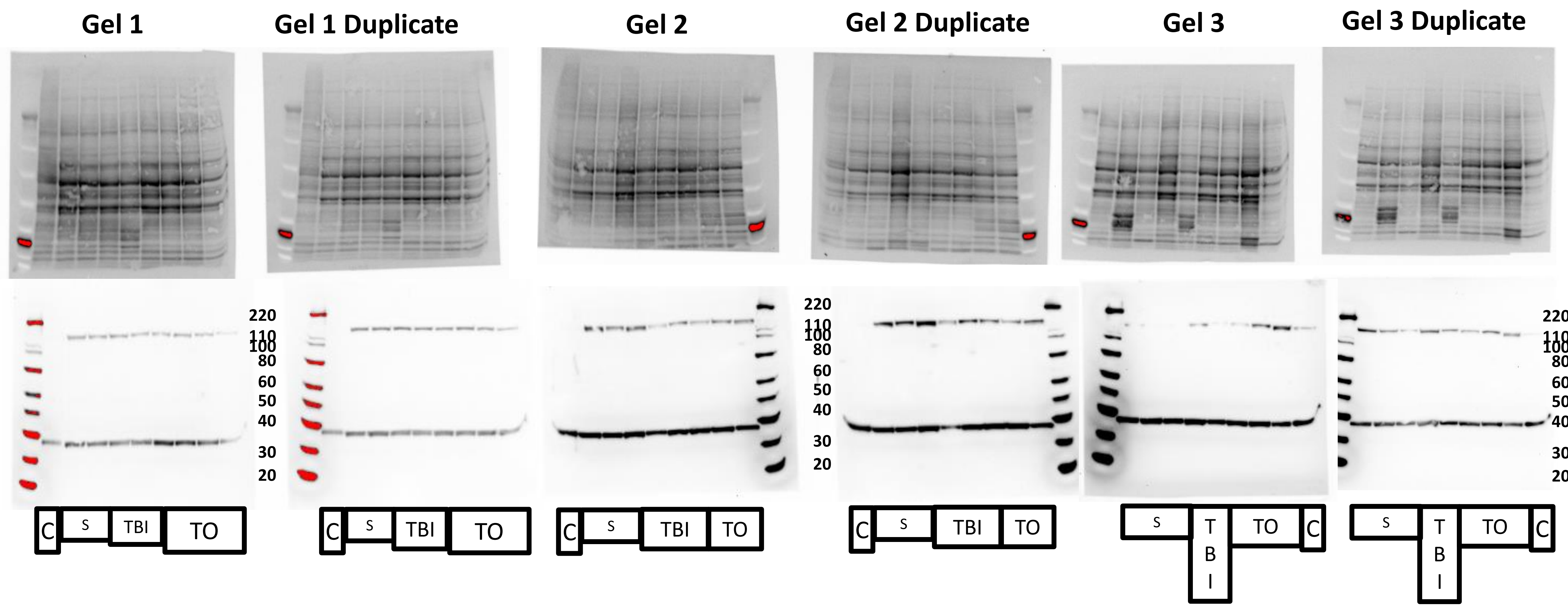

Raw Western Blot Membranes for PERK & eIf2a 7 DPI

Bands obscured by anything we not included in analysis. Duplicates were averaged. Intermembrane calculations were done as described in our previous publication[13]. Top blot = Total Protein, Bottom Blot = Chemiluminescent image of primary antibody (PERK= ~140kDa; eIF2a = ~38). Ladder = Magic Mark. C = Intermembrane Control, H = H_2O (negative), S=SHAM, TBI = Room Air Group, TO = TBI $+O_2$

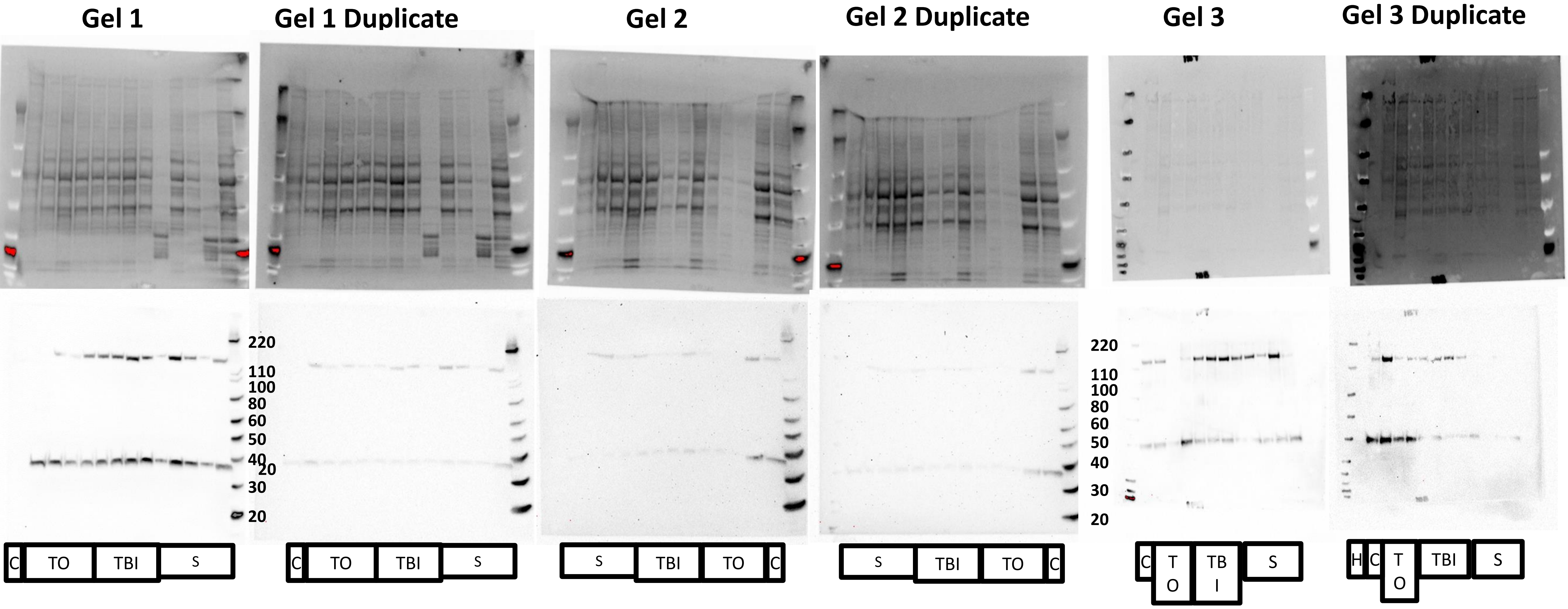

Raw Western Blot Membranes for PERK & eIf2a 30 DPI

Bands obscured by anything we not included in analysis. Duplicates were averaged. Intermembrane calculations were done as described in our previous publication[13]. Top blot = Total Protein, Bottom Blot = Chemiluminescent image of primary antibody (PERK= ~140kDa; eIF2a = ~38). Ladder = Magic Mark. C = Intermembrane Control, H = H_2O (negative), S=SHAM, TBI = Room Air Group, TO = TBI +O_2

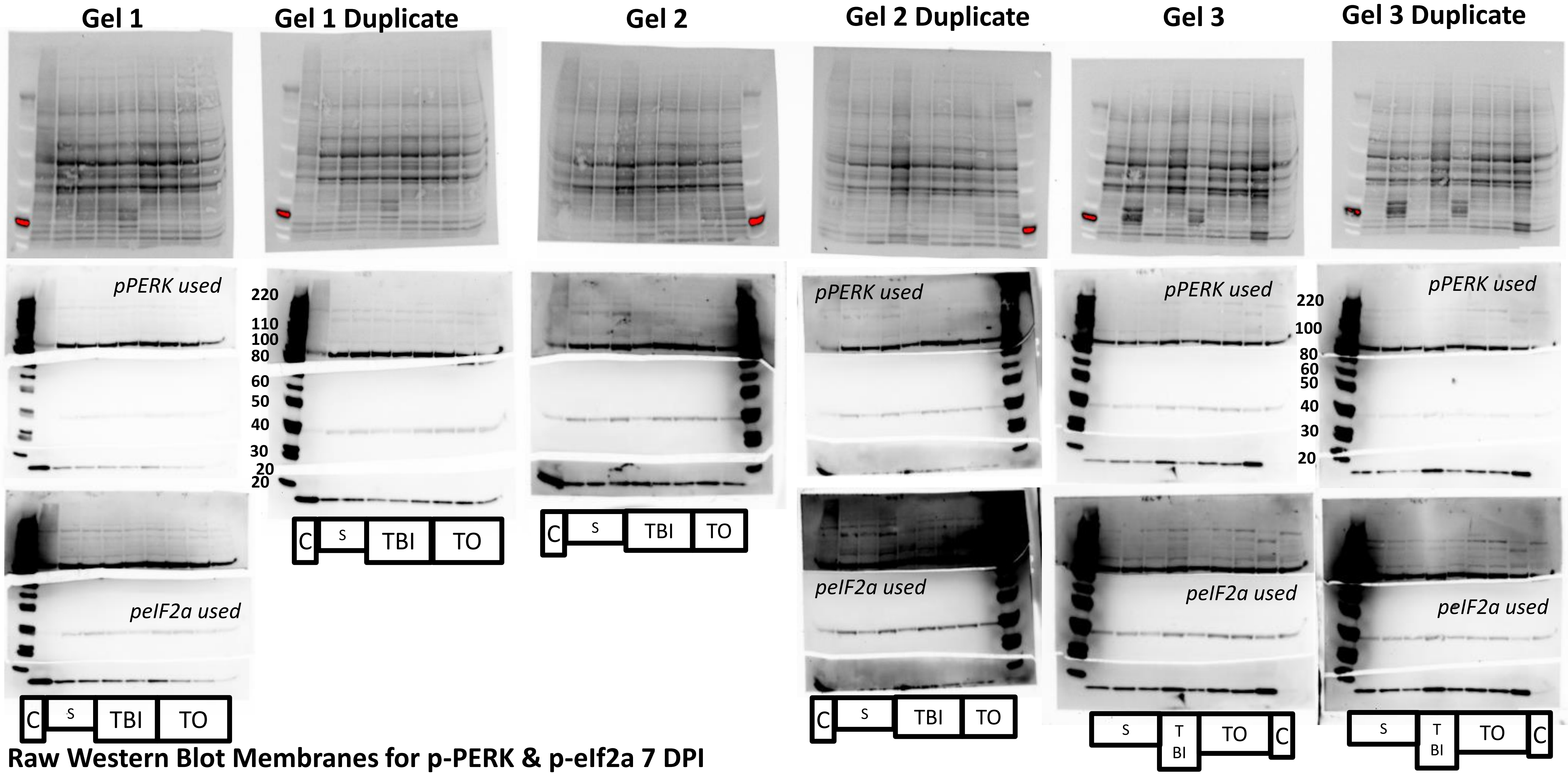

Raw Western Blot Membranes for p-PERK & p-eIf2a 7 DPI

Bands obscured by anything we not included in analysis. Duplicates were averaged. Intermembrane calculations were done as described in our previous publication[13]. This blot was stripped. Exposure used for the appropriate primary antibody is specified on the images. Top blot = Total Protein, Bottom Blot = Chemiluminescent image of primary antibody (PERK (top half)= ~80-110kDa; eIF2a (middle piece)= ~38) The bottom piece was used for another primary antibody that was not included. Ladder = Magic Mark. C = Intermembrane Control, H = H_2O (negative), S=SHAM, TBI = Room Air Group, TO = TBI +O_2

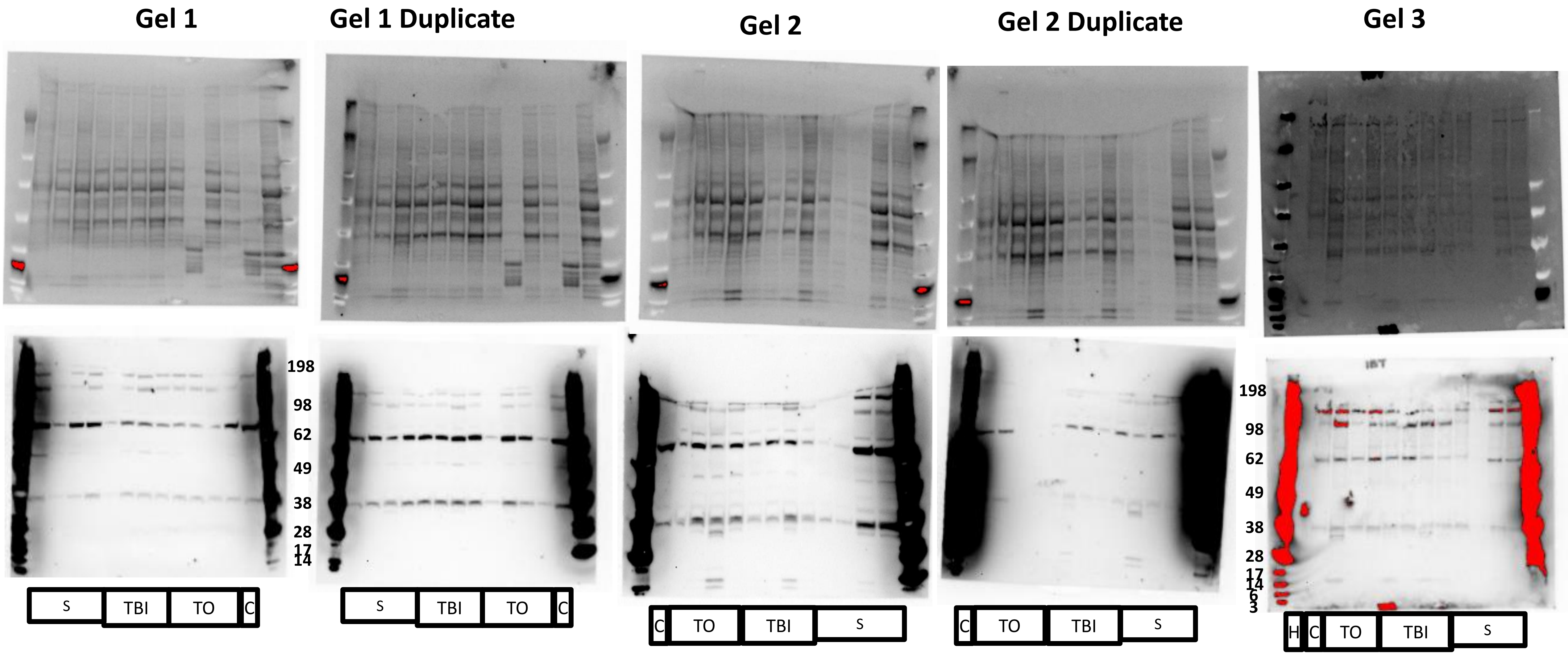

Raw Western Blot Membranes for p-PERK & p-eIf2a 30 DPI

Bands obscured by anything we not included in analysis. Duplicates were averaged. Intermembrane calculations were done as described in our previous publication[13]. This blot was stripped. Top blot = Total Protein, Bottom Blot = Chemiluminescent image of primary antibody (PERK (top half)= ~110-80kDa; eIF2a (middle piece)= ~38) The bottom piece was used for another primary antibody that was not included. Ladder = SeeBluePlus2. C = Intermembrane Control, H = H_2O (negative), S=SHAM, TBI = Room Air Group, TO = TBI +O_2

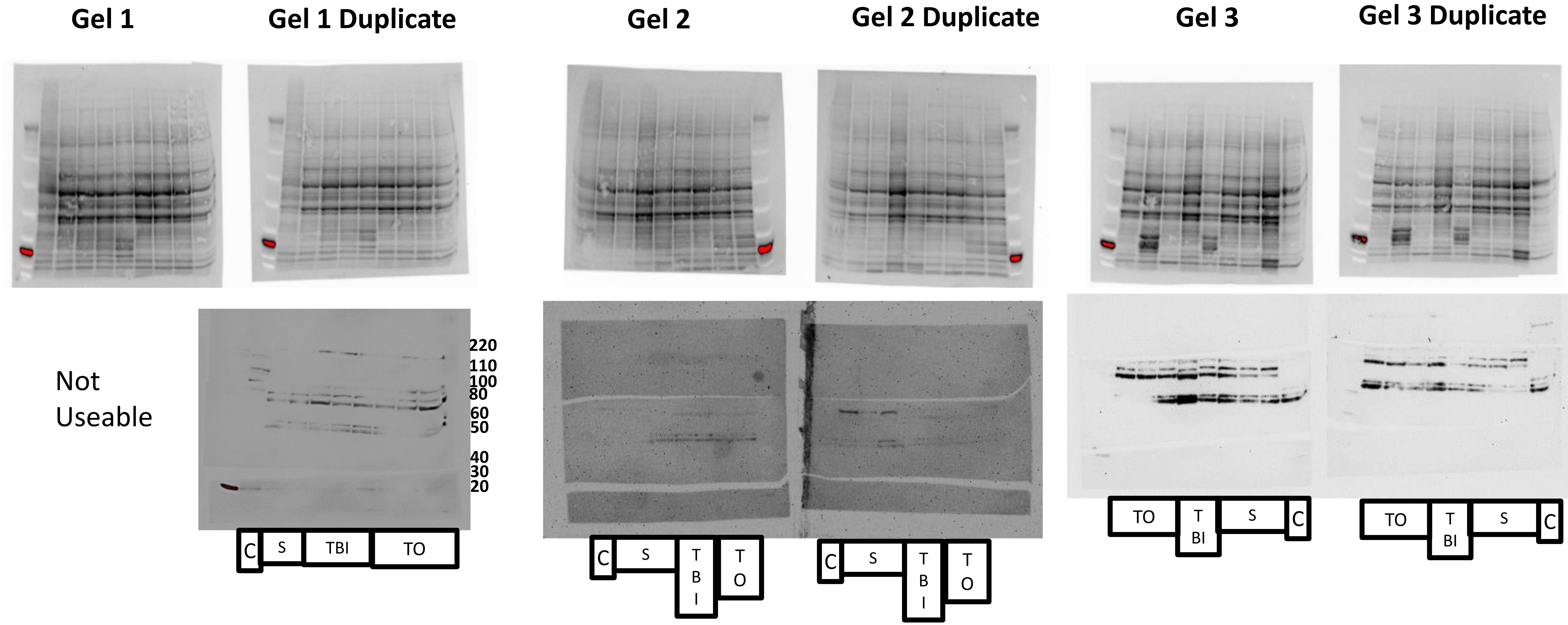

Raw Western Blot Membranes for ATF4 (phos and total) 7 DPI

Bands obscured by anything we not included in analysis. Duplicates were averaged. Intermembrane calculations were done as described in our previous publication[13]. Top blot = Total Protein, Bottom Blot = Chemiluminescent image of primary antibody (ATF4 total = ~42kDa; eIF2a = ~50kDa). Ladder = Magic Mark. C = Intermembrane Control, H = H_2O (negative), S=SHAM, TBI = Room Air Group, TO = TBI +O_2

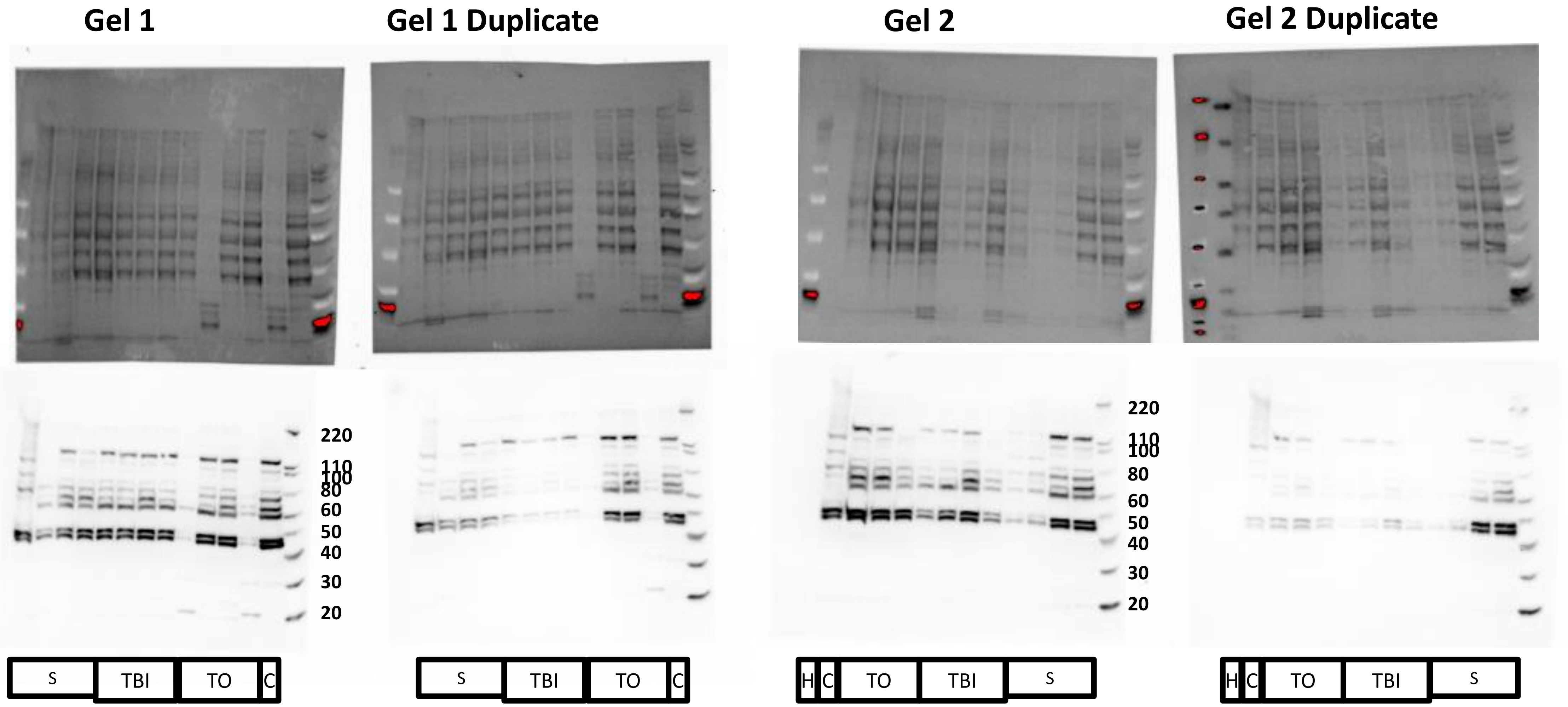

Raw Western Blot Membranes for ATF4 (phos and total) 30 DPI

Bands obscured by anything we not included in analysis. Duplicates were averaged. Intermembrane calculations were done as described in our previous publication[13]. Top blot = Total Protein, Bottom Blot = Chemiluminescent image of primary antibody (ATF4 total = ~42kDa; eIF2a = ~50kDa). Ladder = Magic Mark. C = Intermembrane Control, H = H_2O (negative), S=SHAM, TBI = Room Air Group, TO = TBI +O_2

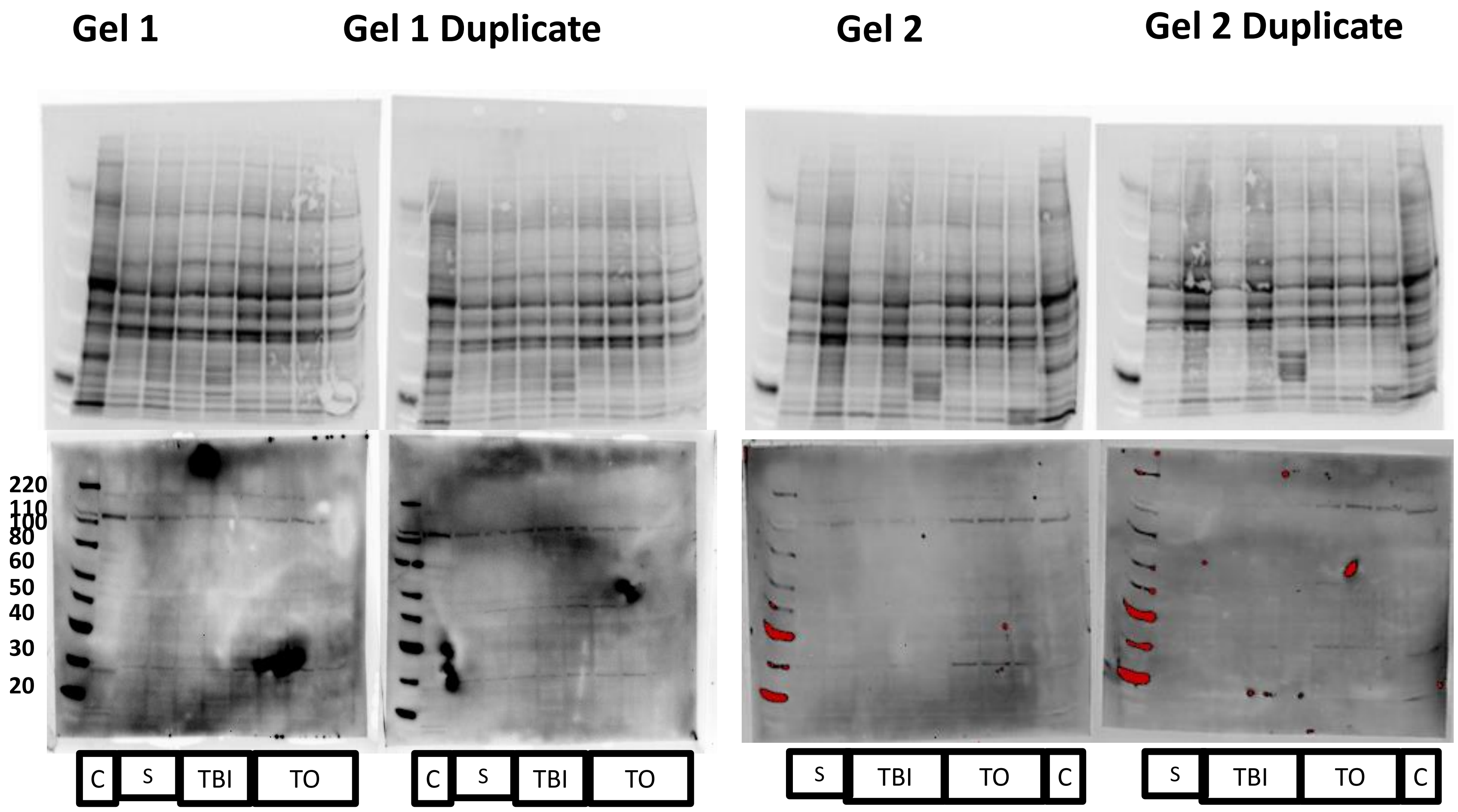

Raw Western Blot Membranes for CHOP 7 DPI

Bands obscured by anything we not included in analysis. Duplicates were averaged. Intermembrane calculations were done as described in our previous publication[13]. Top band (~110kDa) is IRE1, which was stained for at the same time. Top blot = Total Protein, Bottom Blot = Chemiluminescent image of primary antibody (CHOP = ~30kDa). Ladder = Magic Mark. C = Intermembrane Control, H = H_2O (negative), S=SHAM, TBI = Room Air Group, TO = TBI +O_2

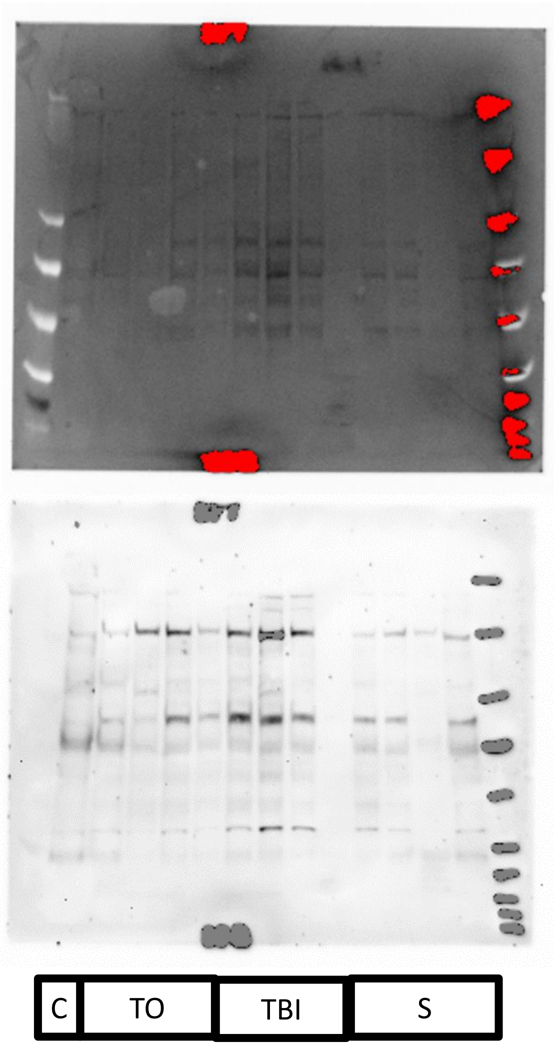

Raw Western Blot Membranes for CHOP 30 DPI

Bands obscured by anything we not included in analysis. Duplicates were averaged. Intermembrane calculations were done as described in our previous publication[13]. Top blot = Total Protein, Bottom Blot = Chemiluminescent image of primary antibody (CHOP = ~30kDa). Ladder = Magic Mark. C = Intermembrane Control, H = H_2O (negative), S=SHAM, TBI = Room Air Group, TO = TBI +O_2

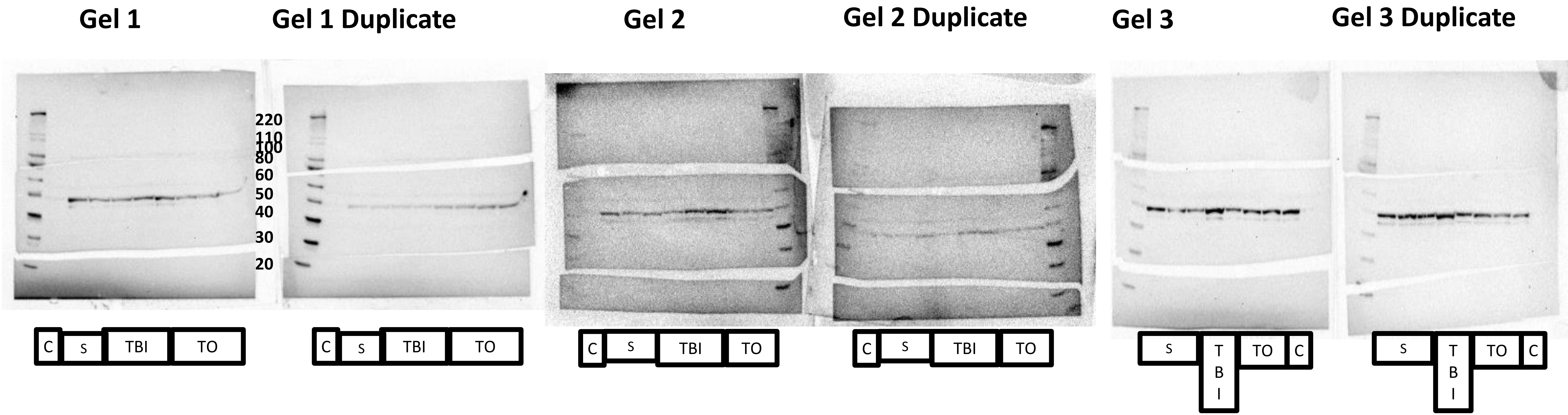

Raw Western Blot Membranes for ERO1La 7 DPI

Bands obscured by anything we not included in analysis. Duplicates were averaged. Intermembrane calculations were done as described in our previous publication[13]. Stripped after p-perk/p-eif2a 7 DPI. (ERO1La= ~50kDa). Ladder = Magic Mark. C = Intermembrane Control, H = H_2O (negative), S=SHAM, TBI = Room Air Group, TO = TBI $+O_2$

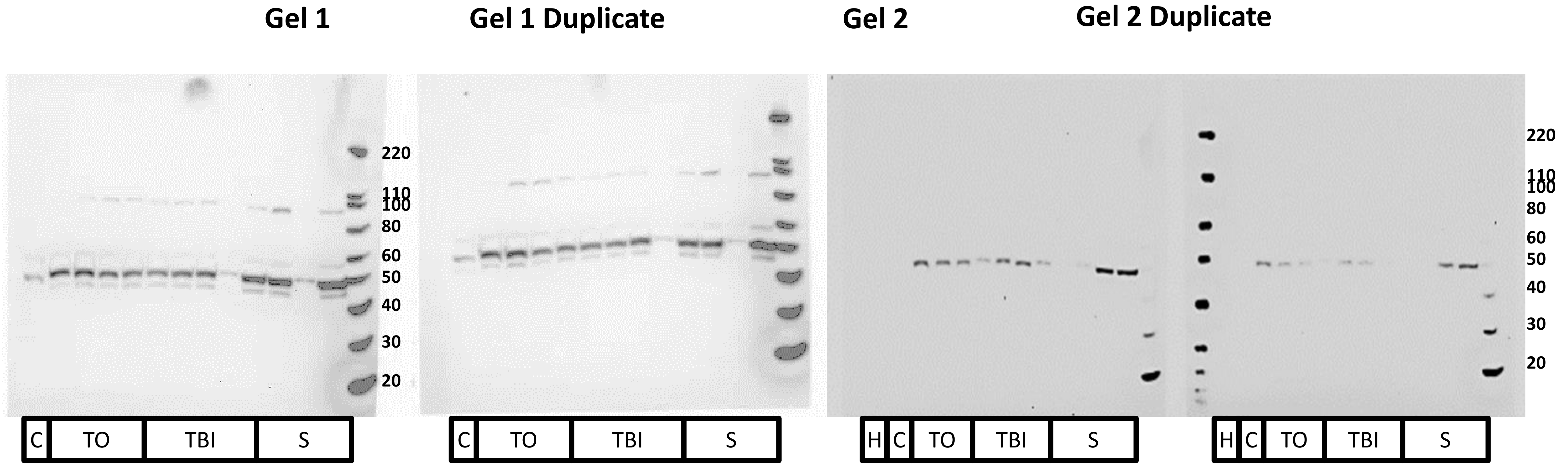

Raw Western Blot Membranes for ERO1La 30 DPI

Bands obscured by anything we not included in analysis. Duplicates were averaged. Intermembrane calculations were done as described in our previous publication[13]. Stripped after XBP1 30 DPI. (ERO1La= ~50kDa). Ladder = Magic Mark. C = Intermembrane Control, H = H_2O (negative), S=SHAM, TBI = Room Air Group, TO = TBI +O_2

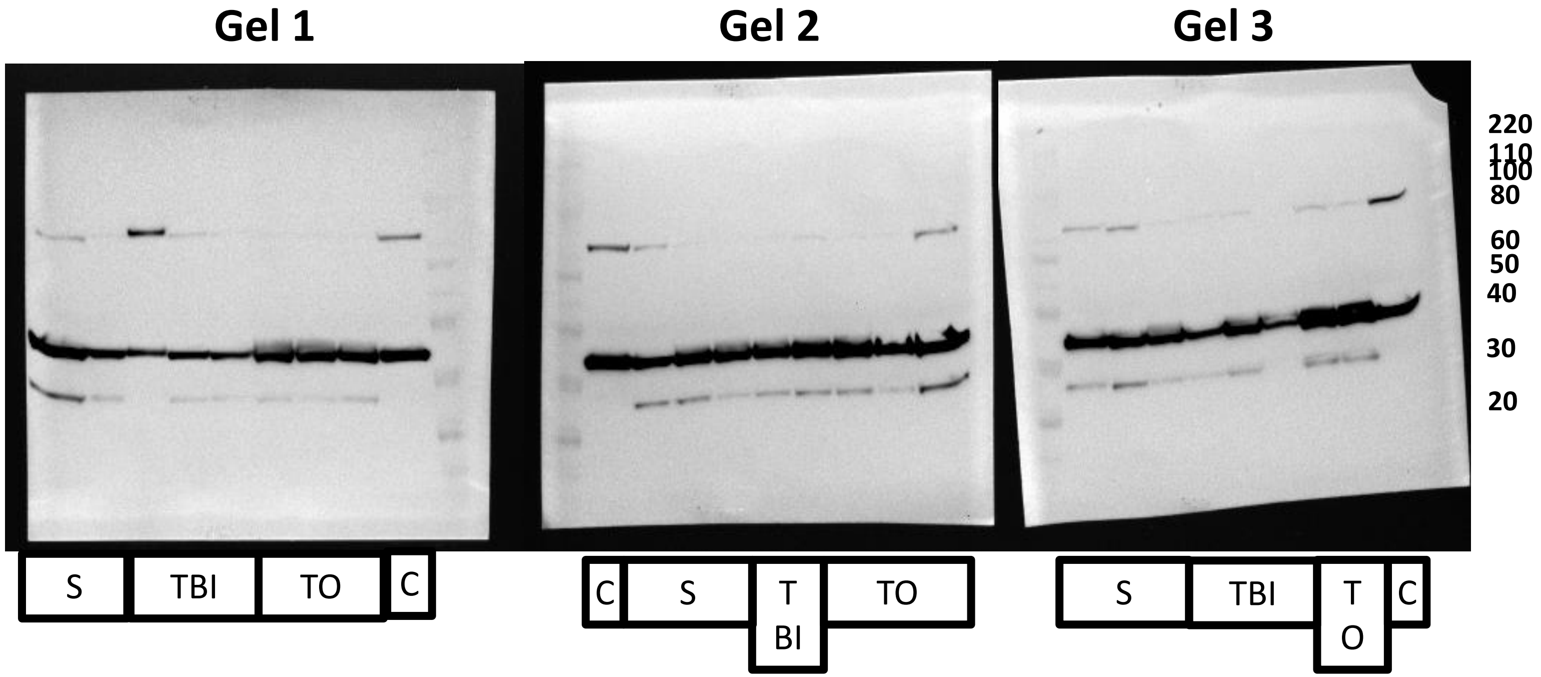

Raw Western Blot Membranes for GADD34 7 DPI

Bands obscured by anything we not included in analysis. Duplicates were averaged. Intermembrane calculations were done as described in our previous publication[13]. Stripped after XBP1. (GADD34= ~73kDa). Ladder = Magic Mark. C = Intermembrane Control, H = H_2O (negative), S=SHAM, TBI = Room Air Group, TO = TBI +O_2

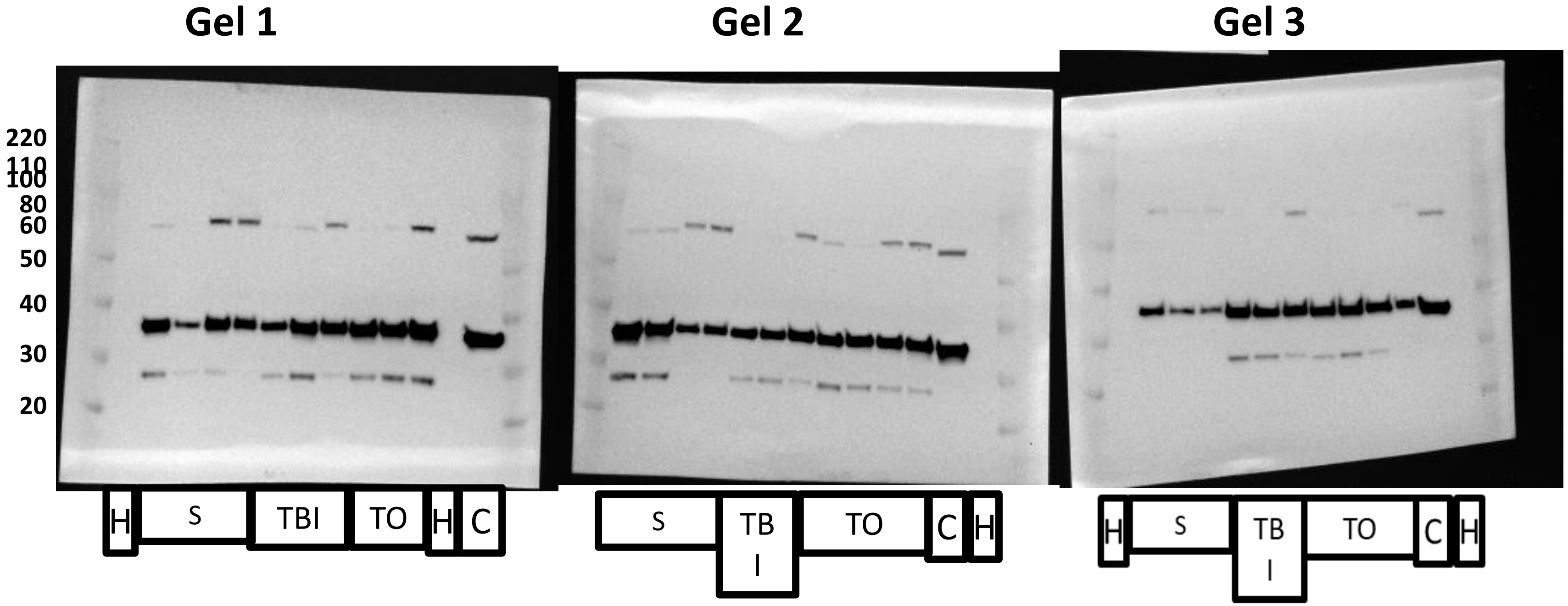

Raw Western Blot Membranes for GADD34 30 DPI

Bands obscured by anything we not included in analysis. Duplicates were averaged. Intermembrane calculations were done as described in our previous publication[13]. Stripped after XBP1. (GADD34= ~73kDa). Ladder = Magic Mark. C = Intermembrane Control, H = H_2O (negative), S=SHAM, TBI = Room Air Group, TO = TBI +O_2
